# *Bartonella* Species in Blood of Immunocompetent Persons with Animal and Arthropod Contact

**DOI:** 10.3201/eid1306.061337

**Published:** 2007-06

**Authors:** Edward B. Breitschwerdt, Ricardo G. Maggi, Ashlee W. Duncan, William L. Nicholson, Barbara C. Hegarty, Christopher W. Woods

**Affiliations:** * North Carolina State University College of Veterinary Medicine, Raleigh, North Carolina, USA,; † Centers for Disease Control and Prevention, Atlanta, Georgia, USA; ‡ Duke University Medical Center, Durham, North Carolina, USA

**Keywords:** Bartonella, culture, PCR, immunocompetent, fatigue, dispatch

## Abstract

Using PCR in conjunction with pre-enrichment culture, we detected *Bartonella henselae* and *B. vinsonii* subspecies *berkhoffii* in the blood of 14 immunocompetent persons who had frequent animal contact and arthropod exposure.

Attempts to isolate *Bartonella* sp. from immunocompetent persons with serologic, pathologic, or molecular evidence of infection are often unsuccessful; several investigators have indicated that *Bartonella* isolation methods need to be improved (*1*–*4*). By combining PCR and pre-enrichment culture, we detected *B. henselae* and *B. vinsonii* subspecies *berkhoffii* infection in the blood of immunocompetent persons who had arthropod and occupational animal exposure.

## The Study

From November 2004 through June 2005, blood and serum samples from 42 persons were tested, and 14 completed a questionnaire, approved by the North Carolina State University Institutional Review Board. Age, sex, animal contact, history of bites, environment, outdoor activity, arthropod contact, travel, and medical history were surveyed. Bacterial isolation, PCR amplification, and cloning were performed by using previously described methods (*5*–*7*). Each blood sample was tested by PCR after direct DNA extraction, pre-enrichment culture for at least 7 days, and subculture onto a blood agar plate (Figure). An uninoculated, pre-enrichment culture was processed simultaneously as a control. Methods used for DNA extraction and conventional and real-time PCR targeting of the *Bartonella* 16S-23S intergenic spacer (ITS) region and heme-binding protein (Pap31) gene have been described (*7*,*8*). Conventional PCR amplicons were cloned with the pGEM-T Easy Vector System (Promega, Madison, WI, USA); sequencing was performed by Davis Sequencing, Inc. (Davis, CA, USA). Sequences were aligned and compared with GenBank sequences with AlignX software (Vector NTI Suite 6.0 (InforMax, Inc., Bethesda, MD, USA) (*7*,*8*). *B. vinsonii* subsp. *berkhoffii*, *B. henselae*, and *B. quintana* antibodies were determined by using a modification of a previously described immunofluorescence antibody assay (IFA) procedure (*9*).

**Figure:**
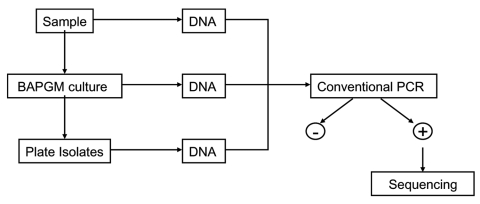
Diagram of sample processing and testing.

Study participants included 12 women and 2 men, ranging in age from 30 to 53 years; all of them reported occupational animal contact for >10 years (Table). Most had daily contact with cats (13 persons) and dogs (12 persons). All participants reported animal bites or scratches (primarily from cats) and arthropod exposure, including fleas, ticks, biting flies, mosquitoes, lice, mites, or chiggers. All participants reported intermittent or chronic clinical symptoms, including fatigue, arthralgia, myalgia, headache, memory loss, ataxia, and paresthesia (Table). Illness was most frequently mild to moderate in severity, with a waxing and waning course, and all but 2 persons could perform occupational activities. Of the 14 participants, 9 had been evaluated by a cardiologist, 8 each by an infectious disease physician or a neurologist, and 5 each by an internist or a rheumatologist. Eleven participants had received antimicrobial drugs.

**Table:** Selected demographic, epidemiologic, and medical information reported by 14 immunocompetent persons infected with *Bartonella henselae* or *B. vinsonii* subsp. *berkhoffii*

Characteristic/symptom	Study participant no.														Total, N = 14
	1	2	3	4	5	6	7	8	9	10	11	12	13	14	
Sex	F	F	F	F	F	F	M	F	F	F	F	F	M	F	
Age, y	51	30	48	44	53	50	32	33	48	53	52	39	52	44	
State of residence	NC	NC	NC	CO	VA	CA	NC	VA	CA	CA	CA	CA	VA	MN	
Occupational animal exposure	V	VtA	AHR	V	V	CR	VtA	VtA	VtA	VtA	VtA	V	WB	WB	
Daily contact with dogs/cats	Y/Y	Y/N	N/Y	Y/Y	Y/Y	Y/Y	Y/Y	Y/Y	Y/Y	Y/Y	Y/Y	Y/Y	N/Y	Y/Y	
Contact with fleas/ticks†	2/1	3/3	4/4	4/4	3/3	2/3	3/3	2/2	4/4	2/4	3/1	3/2	NA/3	4/3	
Self-health assessment‡	CI	CI	II	II	II	CI	CI	CI	CI	II	II	CI	II	CI	
Fatigue	+	+	–	+	+	+	+	+	+	+	+	+	+	+	13
Joint pain	+	+	–	+		+	+	+	–	+	+	+	+	U	10
Difficulty sleeping (insomnia)	+	+	–	–	+	–	+	+	+	+	+	+	–	–	9
Muscle pain	+	+	–	–		U	+	+	–	+	U	+	+	+	8
Difficulty remembering	+	+	–	–	+	–	+	+	+	+	+	–	–	U	8
Loss of sensation or numbness	+	+	+	–		+	–	+	–		+	+	–	U	7
Balance problems	+		–	–	+	+	+	+	–		+	+	–	–	7
Headache	+	+	–	–		+	+	+			–	+	+	U	7
Tremors	+		–	–		–	+	+	–	+	+	+	–	–	6
Irritability	+		–	–		–	+	+	+		+	–	–	+	6
Bowel or bladder dysfunction	+		–	+		+	–	+	–		+	–	+	–	6
Eye pain	+		–	+		+	+	+	–		–	–	–	–	5
Blurred vision	+		–	–		–	+	+	–	+	+	–	–	–	5
Sleepiness	+		–	–		–	+	–	–	+	+	–	–	+	5
Syncope or fainting episodes	+	+	+	–	+	+	–	–	–		–	–	–	–	5
Shortness of breath	+		–	+		–	+	+	–		+	–	–	U	5
Muscle weakness	+		–	–			+		–	+	+	+	–	U	5

*F, female; M, male; NC, North Carolina, CO, Colorado, VA, Virginia; CA, California, MN, Minnesota; V, veterinarian; VtA, veterinary assistant; AHR, animal health researcher; CR, cattle rancher; WB, wildlife biologist; Y, yes; N, no, with respect to the study participant’s daily contact with dogs/cats; CI, chronically ill; II, infrequently ill; +, yes; –, no; blank, no answer reported; U, unknown. †Reported as frequencies and defined as follows: 1, daily; 2, infrequently (weekly); 3, occasionally (monthly); 4, almost never (yearly). ‡Self-health assessment: As part of the questionnaire, study participants were asked to rate their own health status: healthy, infrequently ill, or chronically ill.

When reciprocal titers of >64 were used, 8 persons were seroreactive to *Bartonella* antigens (Appendix Table). *B. henselae* or *B. vinsonii* subsp. *berkhoffii* was detected or isolated from all 14 participants. At the time of initial testing, *Bartonella* DNA was amplified directly from 3 blood samples, from 7 pre-enrichment liquid cultures, and from 4 subculture isolates (Appendix Table). For 5 persons, results of PCR and culture of initial samples were negative. Overall, *Bartonella* DNA was amplified from 11 (28%) of 40 extracted blood samples, 13 (33%) of 40 pre-enrichment cultures, and 5 isolates. For 7 persons, *B. henselae* DNA was amplified at multiple time points. *Bartonella* DNA was never amplified from any PCR control or uninoculated culture control.

By using the ITS target region, 2 distinct *B. henselae* ITS and Pap31 strains were sequenced, *B. henselae* Houston I (HI) (GenBank NC-005956) and *B. henselae* San Antonio 2 (SA2) (GenBank AF369529). Within the noncoding ITS region, *B. henselae* SA2 strains have a 30-bp insertion (ATT GCT TCT AAA AAG ATT GCT TCT AAA AAG) located 518 bases downstream from the 16S gene. Only *B. vinsonii* subsp. *berkhoffii* types I and II were detected (*8*).

## Conclusions

Persistent human infection with *B. bacilliformis* and *B. quintana* has been previously documented, whereas infection with *B. henselae* (cat-scratch disease [CSD]) is generally considered self-limiting (*1*,*2*,*10*). Recently, *B. henselae* DNA was amplified from the blood of a child 4 months after CSD diagnosis (*11*). Our study indicates that *B. henselae* and *B. vinsonii* subsp. *berkhoffii* can induce occult infection in immunocompetent persons and that detection can be enhanced by combining PCR with pre-enrichment culture. Considering only the results from initial blood samples, PCR detected *Bartonella* DNA in 3 samples, all of which were subsequently PCR positive by subculture or enrichment culture. In samples from 5 persons, pre-enrichment was necessary, and in 5 other persons, sequential sampling was necessary to detect *Bartonella* infection. Intermittent bacteremia, as occurs in *B. henselae*–infected cats (*12*), antimicrobial drug administration, low bacterial copy numbers, and low inoculum volume (1 mL) may have contributed to intermittent detection or inability to isolate *Bartonella* spp. from some participant samples. Although our approach is an improvement over historical isolation approaches, our results emphasize ongoing limitations associated with the detection of *Bartonella* infection. Obtaining stable *Bartonella* subcultures (n = 5 in this study) has proven problematic for other specialized laboratories that routinely culture for *Bartonella* spp. (*3*,*4*). To our knowledge, the *B. vinsonii* subsp. *berkhoffii* type II isolate described in our study is the only type II human isolate reported to date (*8*). Various combinations of *B. henselae* and *B. vinsonii* subsp. *berkhoffii* strain types were detected in the same blood sample or sequential blood samples. The coexistence of *B. henselae* genetic variants has been described among primary patient isolates, which suggests that multiple genotypes may emerge within the same person (*13*).

Overall, 57% of persons tested were seroreactive to 1 or all 3 *Bartonella* test antigens. Previous reports from the United States identified a *B. henselae* seroprevalence of 3% in healthy blood donors and a cumulative seroprevalence of 7.1% to both *B. henselae* and *B. quintana* antigens in veterinary professionals (*1*). In this and other studies, serologic test results did not correlate with PCR amplification or isolation results. Antigenic variability among *B. henselae* test strains can cause false-negative IFA results in persons with suspected CSD. Also *B. henselae*, *B. quintana*, or *B. elizabethae* antibodies were not detected in some persons with DNA evidence of active infection (*1*,*3*,*4*).

Animal contact, often to a wide spectrum of domestic and wild animal species, is an obvious consequence of the daily activities of the study population, which is biased by veterinary occupational exposure and by self-selection (volunteer bias). Cats are considered the primary reservoir host for *B. henselae*, whereas coyotes and foxes are considered reservoir hosts for *B. vinsonii* subsp. *berkhoffii* (*1*,*2*,*8*). Detection of *B. vinsonii* subsp. *berkhoffii* in 4 of 5 Californian participants could be related to the high prevalence of bacteremic coyotes in this region as well as to the potential transmission by a tick vector (*1*,*2*). All 14 participants reported frequent arthropod exposure. Although *Bartonella* spp.transmission by ticks has not been proven, several recent studies have identified *Bartonella* DNA in questing ticks, ticks attached to animals, and ticks attached to humans (*1*,*2*,*14*).

Despite reporting chronic or episodic illness, most participants continued to effectively maintain daily professional and personal activities. The symptoms described in the study patients are very similar to those described in a community and hospital-based surveillance study of CSD patients, in whom CSD-associated arthropathy was an uncommon chronic syndrome affecting mostly young and middle-age women (*15*). Our study was initiated to investigate the feasibility of combining PCR with pre-enrichment culture. Prospective studies, with appropriate controls, are needed to characterize the prevalence and clinical relevance of persistent *Bartonella* infection in immunocompetent persons.

## Supplementary Material

Appendix Table Serologic and PCR results from blood collected at multiple time points from 14 persons with frequent animal and arthropod contact
