# Supplementary material for: Bartonella Species in Blood of Immunocompetent Persons with Animal and Arthropod Contact
Source: Emerg Infect Dis. 2007 Jun;13(6):938–41. doi: 10.3201/eid1306.061337 (PMC2792845; doi:10.3201/eid1306.061337)
Supplement: Appendix Table — Serologic and PCR results from blood collected at multiple time points from 14 persons with frequent animal and arthropod contact [file 06-1337_appT-s1.pdf]

Appendix Table. Serologic and PCR results from blood collected at multiple time points from 14 persons with frequent animal and arthropod contact

Participant no./ day sample collected	*Bartonella* PCR result			*Bartonella* IFA reciprocal titers		
	Direct extraction from blood/serum	After 7-d pre-enrichment culture	Blood agar plate isolate	*B. vinsonii berkhoffii*	*B. henselae*	*B. quintana*
Participant 1						
0	Neg	Neg	*B. henselae* H1-like†‡	N/A	N/A	N/A
50	*B. henselae* H1-like†‡	Neg	Neg	32	<32	<32
67	Neg	Neg	Neg	128	32	64
165	Neg	*B. henselae*	Neg	<32	<32	<32
239	Neg	Neg	Neg	N/A	N/A	N/A
299	Neg	Neg	Neg	<32	<32	<32
351	Neg	Neg	Neg	256	64	<32
Participant 2						
0	Neg	Neg	Neg	<32	<32	<32
72	Neg	Neg	Neg	<32	<32	<32
89	*B. henselae* SA2-like†‡	Neg	Neg	<32	<32	<32
106	Neg	*B. henselae* SA2-like†‡	Neg	128	64	64
Participant 3						
0	Neg	*B. henselae* SA2-like†‡	Neg	512	128	128
44	Neg	Neg	Neg	1024	256	256
105	Neg	*B. henselae* SA2-like†‡	Neg	512	256	256
319	Neg	Neg	Neg	512	256	256
Participant 4						
0	Neg	*B. henselae* SA2-like†‡	Neg	64	<32	64
33	Neg	Neg	Neg	N/A	N/A	N/A
Participant 5						
0	*B. vinsonii subsp. berkhoffii* (type II)†‡	Neg	*B. vinsonii subsp. berkhoffii* (type II)†‡	<32	<32	<32
26	*Bh* (SA2-like)†§¶	Neg	Neg	<32	<32	<32
Participant 6						
0	Neg	Neg	Neg	<32	<32	<32
35	*B. henselae*¶	*B. henselae* SA2-like†‡	*B. henselae* SA2-like†¶	<32	<32	<32
147	*B. henselae* SA2-like†¶	Neg	Neg	<32	<32	<32
Participant 7						
0	Neg	*B. henselae* SA2-like†‡, *B. vinsonii subsp. berkhoffii* (type II)†‡	Neg	32	<32	<32
31	Neg	Neg	Neg	128	<32	<32
Participant 8						
0	Neg	*B. henselae*[3]	*B. henselae* SA2-like†¶	<32	<32	<32
25	Neg	Neg	Neg	<32	<32	<32
183	Neg	*B. henselae* H1-like†¶	Neg	<32	<32	<32
215	Neg	Neg	Neg	32	<32	<32
Participant 9						
0	*B. vinsonii subsp. berkhoffii*	*B. vinsonii subsp. berkhoffii*	Neg	N/A	N/A	N/A

	(type I)†‡	(type I),†‡ *B. henselae*‡				
82	Neg	Neg	Neg	256	128	128
Participant 10						
0	*B. vinsonii subsp. berkhoffii* (type I)†¶	Neg	*B. henselae* SA2-like†¶	128	64	128
92	*B. vinsonii subsp. berkhoffii* (Type II)†‡§	Neg	Neg	128	64	128
Participant 11						
0	Neg	Neg	Neg	32	<32	32
103	*B. vinsonii subsp. berkhoffii* (type II)†‡	Neg	Neg	<32	32	32
Participant 12						
0	Neg	Neg	Neg	64	32	32
193	Neg	*B. vinsonii subsp. berkhoffii* (type II)†‡	Neg	256	128	128
Participant 13						
0	Neg	*B. vinsonii subsp. berkhoffii* (type II)†¶	Neg	<32	<32	<32
Participant 14						
0	Neg	Neg	Neg	<32	<32	<32
62	*B. henselae* SA2-like†‡¶	*B. henselae*‡	Neg	<32	<32	<32
146	Neg	Neg	Neg	<32	<32	<32

*IFA, immunofluorescence antibody assay; Neg, negative for *Bartonella* spp. by PCR; NA, serum not available for testing; H1, *B. henselae* Houston 1; SA2, *B. henselae* San Antonio 2.

†Independent PCR from blood and serum identified a *Bartonella spp.*

‡Identified by DNA sequencing.

§Blood source for PCR or sequencing result.

¶Serum source for PCR or sequencing result.
